# Beyond upgrading typologies – In search of a better deal for honey value chains in Brazil

**DOI:** 10.1371/journal.pone.0181391

**Published:** 2017-07-25

**Authors:** Hugo S. de Figueiredo Junior, Miranda P. M. Meuwissen, Ivo A. van der Lans, Alfons G. J. M. Oude Lansink

**Affiliations:** 1 Business Economics Group, Wageningen School of Social Sciences (WASS), Wageningen University, Wageningen, The Netherlands; 2 Accounting Department, School of Economics, Administration, Actuarial Sciences and Accounting, Federal University of Ceará, Fortaleza, Ceará, Brazil; 3 Marketing and Consumer Behaviour Group, Wageningen School of Social Sciences (WASS), Wageningen University, Wageningen, The Netherlands; International Nutrition Inc, UNITED STATES

## Abstract

Selection of value chain strategies by development practitioners and value chain participants themselves has been restricted to preset types of upgrading. This paper argues for an extension of the range of strategy solutions to value chains. An empirical application identifies successful strategies for honey value chains in Brazil for 2015–2020. Strategy and performance indicators were selected using the value chain Structure-Conduct-Performance (SCP) framework. Experts’ opinion was elicited in a Delphi for business scenarios, and adaptive conjoint analysis was used to identify strategies for increasing production growth and local value-added. This study identifies important strategies beyond upgrading typologies, and finds that important strategies differ by performance goal and scenario. The value chain SCP allows searching for promising strategies towards performance–the “better deal”–in an integrated way.

## Introduction

Development organizations have supported value chain interventions to reduce poverty and increase local chain competitiveness [1, 2]. A crucial task in any value-chain-oriented development project is to identify the value chain strategies that are most effective in achieving its desired goals [3, 4]. The scope of an intervention includes adoption of new value chain strategies [5] and attempts to change the enabling environment [6], or both at the same time [7]. Aside from value chain interventions, participants of a value chain may frequently need to change their joint strategies in response to new structural events [8–10]. New structural events may relate to changes in industry structure outside of the control of chain participants.

In this context, the strategic notion of upgrading has been influential: looking for rent-rich opportunities that allow firms to add value to their products through shifts in their capabilities, technologies, and targeted markets. Four types of upgrading were originally identified: product, process, functional, and inter-chain [11]: a) product–moving into more sophisticated products with increased unit value; b) process–achieving a more efficient transformation of inputs into outputs through reorganization of activities or introduction of new technologies; c) functional–acquiring new functions (or abandoning old ones) that increase the skill content of activities; d) inter-chain (or inter-sectoral)–applying competences acquired in one function of a chain into a different sector/chain. In response to empirical evidence from a variety of chains and locations, new upgrading typologies have been suggested that account for vertical and horizontal linkages, and network organizational forms among chain members [12]. A variant of the original typology replaces inter-chain with channel upgrading, which involves diversifying into new buyers or moving into new markets [4]. Another variant adds end-market upgrading, which also relates to the idea of market diversification, and linkages/supply chain upgrading, which relates to strengthening vertical linkages [13]. In addition, the upgrading concept was extended to accommodate enabling environment changes that can impact the outcomes of interventions [14]. Although all value-chain development interventions take place in a certain territory, the upgrading discussion has given little attention to the entire segment of the value chain bounded within a territory [15, 16], and to the local dynamics that shape the chain [17]. This is because upgrading originated from the classic global value chain (GVC) theory [11, 18, 19], which initially focused on bilateral, firm level linkages, and is now evolving to consider relations in larger portions of the chain [20–22]. The challenge to value chain strategy setting has been to select alternatives from the portfolio of the widening concept of upgrading. Upgrading typologies have created shortcuts to the task of identifying chain strategies but may overlook alternative chain strategies. In situations where volume and economies of scale are relevant, for instance, some upgrading paths may not even be the most appropriate to promote local development [23, 24]. The existing literature currently lacks a more general approach, beyond widening the concept of upgrading, to identify the range of strategy options that value chains can choose in different situations.

Complementarily, the importance of territoriality to value chains has been acknowledged by the global production network (GPN) theory, which also gives more emphasis to actors and relationships in global networks [25, 26]. GPN replaces the concept of upgrading with the concepts of value creation and capture [27], acknowledges the possibility of using networks as units of analysis, but remains with firms as units of analysis [28]. Nonetheless, the connection between firm upgrading (or value creation) and local development, in both GPN and GVC theories, has been challenged [24, 29].

**Fig 1:**
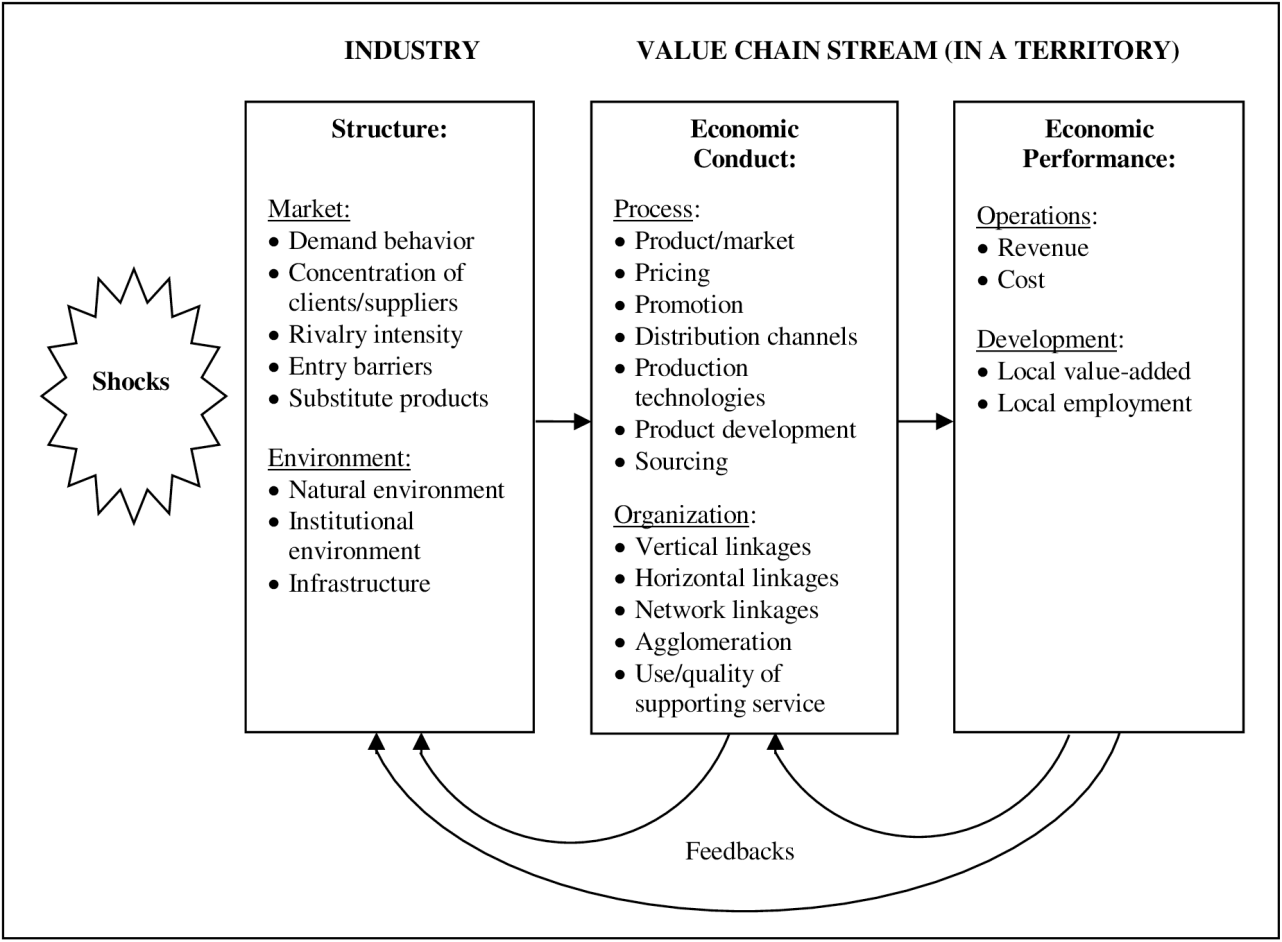
Value chain SCP framework and its categories. Source: [30].

To avoid limiting the range of strategic value chain alternatives to upgrading typologies only, this paper uses the value chain Structure-Conduct-Performance (SCP) framework, developed specifically to address value chain strategies [30]. This framework is an extension of the dynamic SCP framework used by managers to conceive strategies for firms [31, 32], and builds on the ideas from industrial organization economics [33, 34]. In this framework, the unit of analysis is not a firm, but a value chain stream–a segment of a value chain in a territory–competing with streams elsewhere. Accordingly, for a given structure, the performance of a value chain stream can be explained by the conduct of the stream. The value chain SCP framework does not only acknowledge direct interactions, but also feedbacks among structure, conduct and performance, plus the existence of shocks, i.e. significant events that can change the way those interactions take place [30]. In this framework, structure, conduct and performance are explained by their categories. For performance, there are categories related to the operations of the stream, and to local development. For conduct, there are categories related to business process decisions, and to organizational decisions. And for structure, there are categories related to market forces, and to the enabling environment (Fig 1). The value chain SCP framework is expected to provide a way to identify and evaluate strategies (conducts) for value chain streams through an integrated and dynamic assessment of structure, conduct, and performance.

Considering the foregoing discussion, the main objective of this paper is to extend of the range of strategy solutions to value chains beyond upgrading typologies. In the empirical application, likely successful strategies for improving the performance of honey value chain streams are identified using the value chain SCP framework. The remainder of this paper is organized as follows. Section 2 justifies and describes the honey value chain streams chosen for empirical application. Section 3 details the methodology anchored on the value chain SCP framework: the construction of scenarios using a Policy Delphi survey and the conception of strategies using Adaptive Conjoint Analysis with experts. The identification of the most important strategies for each scenario is reported in Section 4, and the discussion of the results and policy conclusions follows in Section 5.

## The honey value chain streams in Brazil

The empirical application focuses on three honey value chain streams in the Northeast of Brazil, all in main honey producing areas of the country. Brazil is among the top 10 honey exporters in the world in volume [35]. Two of the streams are located in Ceará State, one in and around the municipality of Limoeiro do Norte and the other in and around the municipality of Santana do Cariri, and the third stream is located in Piauí State, in and around the municipality of Picos. In 2011, the three streams accounted for 11.2% of Brazilian honey production [36] and 10.2% of the country’s honey exports in volume [37].

Among the streams, Picos has the largest honey production (almost 2.5 thousand t/year), and Santana do Cariri the largest honey export volume (just above 1.0 thousand t/year). The producers are usually small, owning on average between 60 and 100 hives each, and beekeeping is a complementary source of income to crops such as corn, cassava, beans, and to a few livestock such as sheep, goats and cattle. The main local processors either sell bulk honey to the United States and Europe, with the help of international traders sitting in these destinations, or fraction honey to sell in the internal market, with the help of dealers in large cities of the country. Supporting market services in the stream include local technical and managerial assistance, and credit providers. The producers, mostly, sell their honey to the processors that offer the higher prices, in a typical market-based relationship. Special long-term agreements may exist among producers and processors when fair trade and organic certifications are in place. A summary of the figures for the selected streams is presented in Table 1. Further information about the streams and the main structural aspects of the honey business that conformed the chain environment is provided in S1 Appendix, Table A.

**Table 1:** Summary of figures for the selected value chain streams (2011).

		Value Chain Stream	
Figure	Limoeiro do Norte	Santana do Cariri	Picos
Honey production (t)	1,323	843	2,495
Honey exports (t)	541	1,071	661
Main markets	United States	United States and European Union	United States, European Union, Brazil (outside the stream)
Number of producers	1,075	653	1,733
Number of local trade intermediaries^*^	3	2	20
Number of local processors^**^	2	2	3
Average size of producers (number of hives)	69	95	60
Average size of processors (processed volume, t)	274	536	300
Number of technical and managerial assistance providers^***^	2	2	2
Number of credit providers^***^	2	2	2

* No information about their traded volume is available. During the season, there is a fluctuating number of intermediaries commissioned by outside processors.

** Only formal honey processors. Out of the 4 in Picos, only 3 were operating in 2011. Informal honey processors exist, but account for only approximately 1% of total local honey processing in every stream.

*** Number of organizations.

Source: [36]; field interviews.

These streams have been receiving support from governmental and donor agencies since 2007, with the intent to promote local development and provide guidance to improve competitiveness. Qualitative and quantitative evaluations of the strategies deployed by those value chain streams towards improving their performance between 2007 and 2011 have already been carried out [38, 39]. Those strategies included level choices for organic and fair trade honey production, for sales to local processors, for credit and technical assistance offers, among others. The past performance figures for those three value chains are shown in Table 2.

**Table 2:** Performance indicator figures of the selected value chain streams.

		Value Chain Stream		
Category	Indicators	Limoeiro do Norte	Santana do Cariri	Picos
Revenue	Honey production growth 2007–2011 (% per year)	2.7%	4.9%	8.0%
	Honey exports value growth 2007–2011 (% per year)	85.4%	10.6%	52.7%
Local value-added^*^	Honey value-added in all stream steps 2011 per total production (US$/total kg produced)	2.4	3.5	2.6
Local employment	Number of beekeepers’ growth 2007–2011 (% per year)	21%	9%	23%

* Proxy calculated by the difference from honey sales and acquisition costs at each step

Source: [38].

Looking forward, the strategies in this paper were designed to be deployed between 2015 and 2020, aiming at promoting chain competitiveness and local development. Two economic performance indicators were chosen as targets for the streams: production growth of apiculture products and local value-added per unit of production of apiculture products (in the remainder of this paper, these indicators are simply referred to as production growth and local value-added, respectively). Production growth represents the stream’s operational performance, its contribution to the stream’s own competitiveness, whereas local value-added represents the stream’s developmental performance, its contribution to local development. The choice of these indicators was based not only on the diversity of goals that the value chain streams have, but also on the familiarity of the experts with the indicators from a previous analysis [39].

## Methodology

### The Delphi survey

A Policy Delphi survey was applied to identify scenarios for the honey business between 2015 and 2020. The use of scenarios is a powerful tool for strategy development [40], and the Delphi technique is widely applied for the construction of scenarios [41]. The Delphi survey technique usually occurs in multiple interaction rounds with experts [42]. The usefulness of Delphi as a means of eliciting group-based judgments is well acknowledged for establishing exogenous variables–or mostly exogenous variables, such as structural aspects of a business–for other future models [43]. In this study, each scenario was described by a set of relevant events that could occur simultaneously, with each event belonging to only one scenario. Given that total agreement among experts about the inclusion of a certain event in a certain scenario was not required, the less time-consuming Policy Delphi was used. Policy Delphi is a variant of the Delphi technique, which explores the diversity of opinions and in which consensus among experts is not necessary [44].

#### Selection of experts

A set of six local experts (two per stream) was identified, which consisted of consultants (two), academics (one), service providers (one), and business people (two) who had been involved with the honey business for at least 10 years. They were informed about the purpose of the study: to lay the foundation for the development of successful strategies for their streams in the next years. All the experts were interviewed between November 2013 and January 2014 by phone, email, or in person, using a structured questionnaire.

#### Construction of scenarios

The experts were first reminded about the structural aspects of the honey business during the 2007–2011 period, through categorized structural indicators (Table A in S1 Appendix). Then, they were presented with a preliminary list of events, both national and international, which could significantly influence the structure of the business during the period from 2015 to 2020. This list was derived from specialized publications and contained seven events, such as rainfall reduction in the Northeast of Brazil because of global climate change and continuation of the European Union (EU) ban on genetically-modified contaminated honey from general sale. Next, they were asked to add any other events they envisioned as being significant to the list. Finally, they were asked to group the events per their likelihood and their impact on the streams in one of three scenarios: pessimistic, realistic, or optimistic.

Events that are very likely to happen were grouped into the realistic scenario, regardless of their positive or negative impact on the streams. Events that are less likely to happen were grouped into the optimistic scenario when they were judged to have a positive impact on the streams, and into the pessimistic scenario when they were judged to have a negative impact. After the first round, both the initial and added events were consolidated in a complete list, containing the frequency each event appeared in the scenarios. After each round, the frequencies were updated, and the results were informed to the experts in the next round. The Delphi was considered finished when most of the experts agreed, for all events, on their positioning in a certain scenario.

### The adaptive conjoint analysis

Adaptive conjoint analysis (ACA) [45] was used to elicit the opinion of experts on the relative contribution of selected value chain strategies to the future performance of the streams in the period 2015–2020, for each of the three identified scenarios, and to explore the actual trade-offs the experts make [46] among different strategies. In the conjoint analysis set-up, strategies are expressed by strategy levels. ACA was used instead of traditional conjoint analysis because the latter would put too much of a cognitive burden on the experts, due to the enormous number of possible strategies [47].

#### Selection of experts

A set of 15 experts was identified, consisting of consultants (three), academics (four), service providers (five), and business people (three), who had been involved with the honey business for at least five years in the two Brazilian states (ten from Ceará and five from Piauí) where the value chain streams are located. This set included the six experts interviewed in the Delphi survey. The experts constituted most of the population (15 out of 27) of knowledgeable people with diverse professional origins who were available. The same experts were consulted during a previous study on the contribution of strategies to past performance [39].

#### Selection of strategies

A preliminary list (Table A in S2 Appendix) of 22 strategies that could be adopted by the selected value chain streams to increase production growth or local value-added was derived from a qualitative analysis using the value chain SCP framework [38]. The group of 15 experts was offered this list in face-to-face interviews and was asked to add any other strategies they thought necessary, given the scenarios. The interviews took place between February and April 2014. Furthermore, the experts were asked to indicate the strategies they believed would be applicable to each stream for each scenario (Table B in S2 Appendix). The additional strategies were solicited separately for production growth and for local value-added.

For each scenario, the 10 most frequently voted strategies from the preliminary list and the additional, non-overlapping strategies receiving more than three votes were selected, to keep the ACA task feasible for the experts. The final list of selected strategies turned out to be the same for all streams, mainly because the experts believed the streams shared most of the structural characteristics and would be influenced much the same way by the expected scenarios. Subsequently, levels were chosen for the selected strategies for each scenario to allow for the application of the conjoint analysis. These levels were guided by observed past choices [39], by suggestions from the experts, and by the range of feasible choices in any scenario. For instance, the experts believed the maximum percentage of honey production to be sold as fair trade by any stream in any scenario was 40%, so the range of honey sold as fair trade was set between 0 and 40%. The midpoint of 20% was picked as the third level because, in the past, one stream had been able to sell 16% of its production as fair trade. The levels were also restricted to a maximum of four per strategy, with this maximum attained only for strategies with categorical levels, to make the conjoint task feasible for respondents. The resulting ACA task contained 19 different strategies for production growth, 15 in the pessimistic scenario, 14 in the realistic scenario, and 13 in the optimistic scenario. For local value-added, there were 17 different strategies, 16 in the pessimistic scenario, 14 in the realistic scenario, and 14 in the optimistic scenario. For example, the strategy ‘honey certified as fair trade’ was selected for production growth only in the realistic scenario and for local value-added in all scenarios (Table 3).

**Table 3:** Strategies and strategy levels by performance indicator for each scenario.

SCP Category	Strategies	Level 1	Level 2	Level 3	Level 4	Production Growth	Local Value-added
Product/ market	Honey exports (% processed volume)	100%	75%	50%	-	P, R, O	R, O
	Exports to markets other than US and EU (% exported volume)	10%	5%	-	-	P	P
	Honey certified as organic (% production)	75%	25%	0%	-	P, R, O	P, R, O
	Honey certified as fair trade (% production)	40%	20%	0%	-	R	P, R, O
	Honey sold as monofloral (% production)	50%	25%	0%	-	None	P, R, O
	Exploitation of new bee product: propolis	no	yes	-	-	P, R, O	P, R, O
	Exploitation of new bee product: pollen	no	yes	-	-	P, R, O	P, R, O
	Exploitation of new bee product: wax	no	yes	-	-	P, R, O	P, R, O
	Indication of stream origin (% honey production)	50%	25%	0%	-	None	P, R, O
Sourcing	Additional sources of bee forages	none	recovery of original forages	cultivation of new forages	both	P	None
Production technologies	Number of HACCP honey house units per 100 beekeepers	2.00	1.00	0.25	-	P, R, O	P, R, O
	Migratory apiculture (distance from base in km)	800	200	0	-	P	None
	Complementary colony feeding	none	natural feed (pollen and honey)	artificial feed (protein and syrup)	-	P, R, O	None
	Use of alternative vehicles (ex. motorized quad bikes) for collection/ transportation of hives	no	yes	-	-	R	None
	Artificial replacement of queen bees	none	induced division of colonies	introduction of genetically improved queen bee	both	P, R, O	P, R, O
Vertical linkages	Honey sold to local processor (% production)	75%	25%	-	-	O	P, R, O
Horizontal linkages	Resource sharing at production step	no sharing	association^a^	cooperative^b^	-	P	P
Network linkages	Number of chain information exchange events per year in the state	6	4	2	-	None	P, R, O
Agglomeration	Number of hives per beekeeper	200	100	60	-	R, O	None
Quality of supporting services	Technical assistance type	non- specialized^c^	specialized^d^	-	-	P, R, O	P, R, O
Use of supporting services	Technical and managerial assistance coverage (% producers)	90%	50%	25%	-	P, R, O	P, R, O
	Credit coverage (% producers)	75%	25%	10%	-	P, R, O	P

P = Pessimistic scenario; R = Realistic scenario; O = Optimistic scenario

^a^ Group of producers formally organized for sharing equipment, labor, and facilities for honey extraction only

^b^ Group of producers formally organized for sharing equipment, labor, and facilities for honey extraction and sales

^c^ Provided by generalist technicians

^d^ Provided by technicians trained and experienced in apiculture.

Source: Field interviews, prepared by the authors.

#### Conjoint model

The conjoint part-worth model [48] used in this study assumes that the levels of the strategies have an additive contribution to the performance indicators. So, the perceived total contribution, *U*_*i*_, associated with the combination of strategy levels can be expressed as:

$$U_{i}=\sum_{j=1}^{n}u(x_{ij})$$

where *x*_*ij*_ is the level that combination *i* has for strategy *j* (j = 1,…, n) and *u(x*_*ij*_*)* is its contribution. Interaction effects among the selected strategy levels were assumed to be negligible.

In ACA, two types of data (see Section 3.2.4) are used to estimate the *u(x*_*ij*_*)’s* for each expert separately, using Ordinary Least Squares (OLS) regression. The first type consists of so-called self-explicated ratings of attributes and attribute level desirability. The second type consists of so-called graded paired comparisons for combinations of levels from a limited number (two or three) of strategies. The resulting utilities are later calibrated for use in simulations based on likelihood ratings made by each expert [45, 49].

#### ACA questionnaire

The experts were first informed about the structural aspects of the honey business for the period 2007–2011 and about the strategies that most influenced performance during this period, obtained from previous studies [38; 39]. The experts were also informed about the performance indicators. Attention was given to local value-added because the experts were less familiar with its calculation. Scenario orientation followed, in which the experts received a detailed explanation about the scenarios and their use, as recommended by [50].

Data collection took place during individual face-to-face interviews with the experts between September and December 2014, using a dedicated software (ACA Sawtooth Software version 5.1.0). For each scenario, the experts were asked to rate the contribution of the ACA-generated strategy combinations to each performance indicator. The events in each scenario were kept in front of the expert during the ACA process. The procedure was pre-tested with five of the experts to find the best way of acquainting the experts with the ACA task. The individual characteristics of the respondents were not expected to influence their opinion and were therefore not investigated. The size of the sample was also too small to relate the experts’ characteristics to differences in utility estimates.

The ACA questionnaire contained four sections for each performance indicator and scenario. In the so-called self-explicated phase of the interview, entailing Sections I and II, the experts were first asked to rate their preference for each strategy level on a scale from 1 (least preferred) to 7 (most preferred) (Section I statement: please evaluate the following strategy levels as to their contribution to the performance indicator). Subsequently, they were asked to rate, for each strategy, the importance of the differences between the most-preferred and the least-preferred levels on a scale from 1 (not important) to 7 (extremely important) (Section II question: if two strategy sets were acceptable in all other aspects, how important would this difference in strategy levels be to the performance indicator?). In Section III, experts were asked to indicate to what extent they prefer one combination of levels from two or three different strategies over another combination (so-called graded paired comparisons for partial profiles). Pairs of partial profiles were to some extent selected by ACA based on the expert’s ratings in the first two sections. In this graded paired comparison (Section III), each respondent saw pairs of partial profiles and rated them on a scale from 1 (strong preference for left-hand partial profile) to 9 (strong preference for right-hand partial profile) (Section III question: if these two sets of strategies were equal in all other aspects, which one would contribute most to the performance indicator?).

In ACA’s Section IV, respondents were asked to rate the likelihoods that each of five combinations of six strategy levels would give maximum performance on a scale from 0 (definitely would not contribute) to 100 (definitely would contribute). The respondents’ estimate of maximum performance was elicited by asking for the maximum performance indicator value that they consider reasonable for 2015–2020 period, as a percentage of the past value (2007–2011 production growth or 2011 local value-added) for all streams, on average. Percentages of past indicators were used because it was expected to be more difficult, even for the experts, to provide answers about future performance in absolute values. The reference to a maximum possible value was included to maintain the original set-up of ACA’s Section IV, in which a scale with more objective anchors is typically used. Section IV ratings were used for re-scaling (by means of OLS regression) the more or less arbitrarily scaled Section I/II/III contributions to logit transformed likelihood ratings [49]. The internal predictive validity (consistency) of the Section I/II/III utilities was measured in terms of the squared multiple correlations (R^2^) from these regressions [51]. In addition, the consistency and the absolute agreement among experts were measured by the two-way random intraclass correlation coefficient of the utilities [52].

#### Further analysis of ACA utilities

Relative strategy importances were derived from the ACA-estimated individual utilities for each performance indicator in each of the three scenarios. That is, for each strategy, the difference between the utility of the most preferred and the least preferred level was expressed in terms of percentages [53, 54]. These percentages, as well as the utilities of each strategy level obtained from the experts, were averaged for each indicator in each scenario. This procedure allowed the identification of the most important strategies and the most preferred strategy level, for each indicator in each scenario.

## Results

### Scenarios for the honey business

No experts dropped out during the Delphi study, which was concluded with a total of 16 relevant events distributed in three non-overlapping 2015–2020 scenarios for the honey business. Each event was placed in only one of the scenarios. The Delphi was concluded after two rounds, with an agreement of at least two thirds of the experts about the placing of an event in a scenario (Table 4). This level of agreement does not reflect the likelihood of the scenarios. By definition, the most likely events are all grouped in the realistic scenario (thus the most likely scenario). All disagreements among the experts referred to the likelihoods of the events, not to the signs of the impacts.

**Table 4:** Placement of events in the 2015–2020 honey business scenarios and the level of agreement amongst experts about the placement.

Scenario	Event	Agreement (%)
Pessimistic	1) Rainfall reduction in the Northeast of Brazil as a consequence of global climate change [55];	100%
	2) Spread of Colony Collapse Disorder (CCD) to Northeast of Brazil;	100%
	3) Return of Chinese honey to the United States (US) market (lift of antidumping import duty [56], expected to continue until at least 2017), which is the main destination of stream exports (above 80% of volume);	67%
	4) Withdraw of donors’ support to the Picos value chain stream;	83%
	5) New ban of Brazilian honey from European Union (EU) market.	100%
Realistic	1) Continuation of EU ban [57] on genetically-modified contaminated honey from general sale (not identified as Genetically Modified Organisms–GMOs) starting 2012;	100%
	2) Growth of honey demand in the external market;	100%
	3) Continuation of the advance of agriculture over bee forages in Argentina;	83%
	4) Continuation of bee deaths in the US (by CCD);	100%
	5) Growth of honey exports from African countries (e.g. Ethiopia);	83%
	6) Prohibition of exporting, by the Brazilian Ministry of Agriculture or the Brazilian Sanitary Inspection Service, of honey extracted in honey houses without the accreditations Good Manufacturing Practices (GMP) and Hazard Analysis and Critical Control Points (HACCP).	67%
Optimistic	1) Continuous growth of national honey consumption (in contrast to 2007–2011 period);	67%
	2) Equity partnerships among local honey processors and global traders (e.g., as occurred with two major processors in Santana do Cariri at the end of 2011);	67%
	3) Devaluation of Brazilian Real in relation to the American dollar, favoring Brazilian exports;	83%
	4) Growth of direct state government subsidies to honey producers in Ceará;	83%
	5) Advance of bee forages over areas previously occupied by traditional and diminishing rural activities like extensive cattle raising and non-irrigated agriculture in the Northeast of Brazil.	83%

Source: Delphi questionnaire with experts.

### Effects of strategies

For the production growth indicator, the mean strategy-level utilities and the relative strategy importances were calculated for each scenario (see S1–S6 Tables under Supporting Information for detailed calculations). For each strategy in a certain scenario, the level with the highest utility is preferred by the experts (Table 5). For instance, exporting at 75% of the processed volume was the preferred level for the strategy ‘honey exports’ in all scenarios.

**Table 5:** Mean strategy-level utilities for production growth by scenario.

Strategy	Level	Utilities by Scenario*		
		Pessimistic	Realistic	Optimistic
Honey exports (% processed volume)	100%	-0.185	-0.014	-0.071
	75%	*0*.*201*	*0*.*187*	*0*.*142*
	50%	0.041	-0.119	0.003
Exports to markets other than US and EU (% exported volume)	10% 5%	*0*.*291* -0.252	- -	- -
Honey certified as organic (% production)	75%	*0*.*196*	*0*.*302*	*0*.*316*
	25%	0.111	0.057	0.075
	0%	-0.249	-0.306	-0.316
Honey certified as fair trade (% production)	40%	-	0.231	-
	20%	-	0.041	-
	0%	-	-0.219	-
Exploitation of new bee product: propolis	no	-0.132	-0.177	-0.223
	yes	*0*.*171*	*0*.*212*	*0*.*273*
Exploitation of new bee product: pollen	no	-0.086	-0.167	-0.184
	yes	*0*.*124*	*0*.*203*	*0*.*234*
Exploitation of new bee product: wax	no	-0.161	-0.195	-0.248
	yes	*0*.*199*	*0*.*231*	*0*.*297*
Additional sources of bee forages	none	-0.508	-	-
	recovery of original forages	0.242	-	-
	cultivation of new forages	0.099	-	-
	both	*0*.*245*	-	-
Number of HACCP honey house units per 100 beekeepers	2.00 1.00	0.097 *0*.*177*	*0*.*289* 0.163	*0*.*299* 0.174
	0.25	-0.217	-0.398	-0.399
Migratory apiculture (distance from base, km)	800	0.100	-	-
	200	*0*.*211*	-	-
	0	-0.253	-	-
Complementary colony feeding	none	-0.426	-0.383	-0.470
	natural feed (pollen/honey)	*0*.*267*	*0*.*258*	*0*.*350*
	artificial feed (protein/syrup)	0.217	0.179	0.194
Use of alternative vehicles for collection/transportation of hives	no yes	- -	-0.115 *0*.*150*	- -
Artificial replacement of queen bees	none	-0.471	-0.510	-0.502
	induced division of colonies	0.126	0.096	0.033
	introduction of genetically improved queen bee	0.175	0.214	0.178
	both	*0*.*247*	*0*.*273*	*0*.*390*
Honey sold to local processor (% production)	75%	-	-	0.338
	25%	-	-	-0.289
Resource sharing at production step	no sharing	-0.467	-	-
	association	0.168	-	-
	cooperative	*0*.*356*	-	-
Number of hives per beekeeper	200	NA	0.132	0.230
	100	NA	0.129	0.173
	60	NA	-0.207	-0.328
Technical assistance type	non-specialized	-0.359	-0.385	-0.370
	specialized	*0*.*397*	*0*.*420*	*0*.*420*
Technical and managerial assistance coverage (% producers)	90% 50%	*0*.*307* -0.043	*0*.*405* -0.028	*0*.*381* 0.064
	25%	-0.206	-0.323	-0.370
Credit coverage (% producers)	75%	*0*.*257*	*0*.*309*	*0*.*317*
	25%	-0.006	0.011	0.060
	10%	-0.193	-0.266	-0.302
Consistency of experts, mean of ACA model R^2^		0.811	0.853	0.881
Intraclass correlation coefficient, α	-	0.953	0.953	0.939

* The most preferred level per strategy in each scenario is shown in italics.

No entry when a strategy is not included in a scenario.

Source: Field interviews, prepared by the authors.

The preferences for the strategy levels changed across the scenarios for production growth, however the most preferred level was generally stable across the scenarios. The only exception was the number of HACCP honey houses per 100 beekeepers. Having one HACCP honey house unit per 100 beekeepers was the preferred level in the pessimistic scenario but became less preferred than two HACCP honey house units per 100 beekeepers in the realistic and optimistic scenarios. The differences in the utilities of the levels for this strategy in each of the scenarios was large, meaning that the experts had similar views about the effects of the strategy levels: in scenarios with growing demand and qualified competition, having more certified capacity would contribute more to production growth. In eight of the remaining strategies, the utility of the preferred level increased as the scenario shifted from pessimistic to optimistic.

For each scenario, the most important strategies for production growth were also identified (Table 6). In the pessimistic scenario, defensive strategies such as ‘additional sources of bee forages’ and ‘resource sharing at production step’ were among the top three strategies in importance (%). In the optimistic scenario, ‘number of hives per beekeeper’ was among the top three. ‘Complimentary colony feeding’, a productivity improvement strategy, rated among the top three strategies in all scenarios. The three most important strategies accounted for approximately 30% of the total importance in each scenario.

**Table 6:** Relative importance of strategies for production growth by scenario.

	Relative importance by scenario (%)*		
Strategy	Pessimistic	Realistic	Optimistic
Honey exports (% processed volume)	4.9	5.6	5.7
Exports to markets other than US and EU (% exported volume)	5.4	-	-
Honey certified as organic (% production)	6.3	7.9	7.8
Honey certified as fair trade (% production)	-	6.7	-
Exploitation of new bee product: propolis	4.5	5.0	5.3
Exploitation of new bee product: pollen	4.7	4.6	5.2
Exploitation of new bee product: wax	4.6	5.2	5.9
Additional sources of bee forages	**9.8**	-	-
Number of HACCP honey house units per 100 beekeepers	5.2	8.4	8.7
Migratory apiculture (distance from base, km)	7.6	-	-
Complementary colony feeding	**9.5**	**9.9**	**11.5**
Use of alternative vehicles for collection/transportation of hives	-	4.0	-
Artificial replacement of queen bees	9.1	**10.2**	**10.5**
Honey sold to local processor (% production)	-	-	6.2
Resource sharing at production step	**9.4**	-	-
Number of hives per beekeeper	-	7.4	**10.0**
Technical assistance type	7.6	**8.9**	8.3
Technical and managerial assistance coverage (% producers)	6.1	8.6	7.8
Credit coverage (% producers)	5.4	7.7	7.2
Total	100	100	100

* The relative importances of the three strategies with the highest relative importances per scenario are shown in bold.

No entry when a strategy is not included in a scenario.

Source: Field interviews, prepared by the authors.

For the local value-added indicator, the mean strategy-level utilities and the relative strategy importances were also calculated for each scenario (see S7–S12 Tables under Supporting Information for detailed calculations). For each strategy in a certain scenario, the level with the highest utility is again the preferred level by the experts (Table 7). For instance, exporting 50% of the processed volume was the preferred level for the strategy ‘honey exports’ in the realistic scenario whereas exporting 75% of the processed volume was the preferred level for the strategy ‘honey exports’ in the optimistic scenario. This strategy ‘honey exports’ was not considered in the pessimistic scenario.

**Table 7:** Mean strategy-level utilities for local value-added by scenario.

Strategy	Level	Utilities by Scenario*		
		Pessimistic	Realistic	Optimistic
Honey exports (% processed volume)	100%	-	-0.119	-0.098
	75%	-	0.091	*0*.*218*
	50%	-	*0*.*107*	-0.031
Exports to markets other than US and EU (% exported volume)	10% 5%	*0*.*257* -0.223	- -	- -
Honey certified as organic (% production)	75%	*0*.*397*	*0*.*426*	*0*.*513*
	25%	0.089	0.025	0.080
	0%	-0.435	-0.372	-0.504
Honey certified as fair trade (% production)	40%	*0*.*302*	*0*.*323*	*0*.*423*
	20%	0.134	0.101	0.129
	0%	-0.385	-0.344	-0.463
Honey sold as monofloral (% production)	50%	*0*.*246*	*0*.*207*	*0*.*262*
	25%	0.127	0.154	0.134
	0%	-0.322	-0.282	-0.308
Exploitation of new bee product: propolis	no yes	-0.210 *0*.*244*	-0.238 *0*.*291*	-0.188 *0*.*247*
Exploitation of new bee product: pollen	no	-0.101	-0.201	-0.182
	yes	*0*.*135*	*0*.*253*	*0*.*241*
Exploitation of new bee product: wax	no	-0.200	-0.186	-0.162
	yes	*0*.*234*	*0*.*238*	*0*.*221*
Indication of stream origin (% honey production)	50%	*0*.*348*	*0*.*273*	*0*.*335*
	25%	-0.012	0.086	0.118
	0%	-0.286	-0.281	-0.364
Number of HACCP honey house units per 100 beekeepers	2.00	*0*.*185*	*0*.*262*	0.120
	1.00	0.072	0.110	*0*.*162*
	0.25	-0.206	-0.293	-0.194
Artificial replacement of queen bees	none	-0.266	-0.196	-0.205
	induced division of colonies	0.068	0.039	0.077
	introduction of genetically improved queen bee	*0*.*176*	*0*.*142*	0.106
	both	0.090	0.121	*0*.*140*
Honey sold to local processor (% production)	75%	*0*.*289*	*0*.*400*	*0*.*343*
	25%	-0.255	-0.347	-0.283
Resources sharing at production step	no sharing	-0.351	-	-
	association	0.115	-	-
	cooperative	*0*.*287*	-	-
Number of information exchange events per year in the state	6	0.064	*0*.*072*	0.032
	4	*0*.*072*	0.056	*0*.*114*
	2	-0.085	-0.048	-0.058
Technical assistance type	non-specialized	-0.276	-0.336	-0.227
	specialized	*0*.*310*	*0*.*389*	*0*.*286*
Technical and managerial assistance coverage (% producers)	90%	*0*.*305*	*0*.*325*	*0*.*313*
	50%	0.038	0.054	-0.008
	25%	-0.293	-0.299	-0.216
Credit coverage (% producers)	75%	*0*.*200*	-	-
	25%	0.024	-	-
	10%	-0.173	-	-
Consistency of experts, mean of ACA model R^2^		0.800	0.809	0.820
Intraclass correlation coefficient, α	-	0.943	0.950	0.953

* The most preferred levels per strategy in each scenario are shown in italics.

No entry when a strategy is not included in a scenario.

Source: Field interviews, prepared by the authors.

Changes in the preferences for strategy levels were also observed across the scenarios for local value-added. For four strategies, these changes were such that a strategy level was no longer preferred (‘honey exports’, ‘number of HACCP house units per 100 beekeepers’, ‘artificial replacement of queen bees’, and ‘number of information exchange events per year in the state’). The difference in the utilities of the second-most preferred and the most preferred strategy level was small for each scenario. For example, exporting 50% of the processed volume was the preferred strategy level in the realistic scenario (with utility 0.107), compared to the 75% strategy level (with utility 0.091) and the 100% strategy level (with utility -0.119). The difference in utilities of the 50% and 75% levels was small in the realistic scenario. This means that the experts were not completely sure about the difference in the effects of the two most-preferred strategy levels in the realistic scenario. In the optimistic scenario, the 50% strategy level was clearly preferred, with utility 0.218 versus utility -0.031 for the 50% level and utility -0.098 for the 100% strategy level. This pattern of perceptions among experts contributed to none of these four strategies being among the most important in any of the scenarios.

For each scenario, the most important strategies for local value-added were also identified (Table 8). In the pessimistic scenario, ‘resource sharing at production step’ was among the top three strategies whereas in the realistic scenario, ‘honey sold to local processor’ was the most important strategy. In the optimistic scenario, a less traditional product-differentiating strategy, ‘indication of stream origin’, was among the top three. Well-known product-differentiating strategies such as ‘honey certified as organic’ and ‘honey certified as fair trade’ rated among the top three in all scenarios. The utilities of the three most important strategies together accounted for 25% to 32% of the total utility in each scenario.

**Table 8:** Relative importance of strategies for local value-added by scenario.

Strategy	Relative importance by scenario (%)*		
	Pessimistic	Realistic	Optimistic
Honey exports (% processed volume)	-	5.9%	6.6%
Exports to markets other than US and EU (% exported volume)	5.2%	-	-
Honey certified as organic (% production)	**9.6%**	**9.6%**	**12.4%**
Honey certified as fair trade (% production)	**8.1%**	**8.8%**	**10.3%**
Honey sold as monofloral (% production)	6.6%	7.5%	7.7%
Exploitation of new bee product: propolis	5.1%	6.0%	5.3%
Exploitation of new bee product: pollen	3.7%	6.3%	5.2%
Exploitation of new bee product: wax	4.9%	5.1%	4.7%
Indication of stream origin (% honey production)	6.7%	7.2%	**9.0%**
Number of HACCP honey house units per 100 beekeepers	5.1%	7.1%	5.8%
Artificial replacement of queen bees	6.2%	5.8%	6.8%
Honey sold to local processor (% production)	6.2%	**10.5%**	7.7%
Resources sharing at production step	**8.0%**	-	-
Number of information exchange events per year in the state	6.0%	4.6%	5.1%
Technical assistance type	6.4%	8.4%	6.4%
Technical and managerial assistance coverage (% producers)	6.8%	7.3%	6.9%
Credit coverage (% producers)	5.6%	-	-
Total	100%	100%	100%

* The relative importances of the three strategies with the highest relative importances per scenario are shown in bold.

No entry when a strategy is not included in a scenario.

Source: Field interviews, prepared by the authors.

Comparing the results of the two performance indicators, it is noteworthy that none of the top three strategies for production growth were among the top three for local value-added. As long as performance goals are not the same, it is expected that the strategies to reach them may also be different. Furthermore, consistency among experts was lower for local value-added than for production growth in all scenarios, as the experts were less familiar with the local value-added indicator.

Overall, the mean R^2^ from the OLS regressions of the Section IV likelihood ratings on the Section I/II/III combined utilities was around 0.80, showing the high internal validity of the individual-level utilities in all scenarios. The intraclass correlation coefficient of utilities was also high, above 90% in all scenarios, expressing a strong agreement across experts in all scenarios (Tables 5 and 7).

## Discussion and conclusions

The results of the analysis can be used for the practical implementation of the strategies. Given that promising strategies vary according to performance goals, value chain streams are advised to first prioritize a performance goal. Then, for the prioritized performance goal, implement the strategies in the order of importance for the realistic scenario, which is the most likely scenario by definition. If the situation changes, for example from realistic to pessimistic during the implementation process, the order of importance of the strategies in the pessimistic scenario should then be followed.

For the three studied streams, during the period 2015–2020, in the realistic scenario, the most promising strategies are ‘artificial replacement of queen bees through both division of colonies and artificial replacement of queen bees’ for ‘production growth’, and ‘honey sold to local processor at the 75% level of production’ for ‘local value-added’. If any stream is already at the level of those strategies, it should go on to implement the strategies that are next in perceived importance. For ‘production growth’, this would mean starting to implement ‘both recovery of original forages and cultivation of new forages as additional sources of bee forages’ and ‘resource sharing at production step as a cooperative’. For ‘local value added’, this would also mean starting to implement ‘resource sharing at production step as a cooperative’.

In practice, the scenarios may not be mutually exclusive, and an event that was expected to be less likely to occur, may happen. The strategies that are more influenced by this particular event could then be pursued. For instance, ‘rainfall reduction as a consequence of global climate change’ was considered less likely to occur and some strategies associated with this event (for instance, ‘additional sources of bee forages’ and ‘migratory apiculture’) were considered relevant in the pessimistic scenario only. However, should this less likely event occur, these strategies should be considered. This shows how the results can be used to find appropriate strategies if an unlikely event occurs. A new conjoint study involving new scenarios may therefore not always be necessary to identify the importance of a specific strategy should an unlikely event occur.

In addition to the significant events that shape the structure of the honey business in each scenario, feedbacks from the performance of the streams to conduct and to structure are expected. In a 5-year planning horizon, however, the effects of those feedbacks at the national or world level are not expected due to the small size of the selected streams. At the local level, however, feedback effects may be relevant and should be monitored by the chain participants as the streams implement and evaluate their strategies.

Although the three honey streams in this study are located relatively close to each other and the group of strategies is the same across the streams, there may be relevant differences in their strategy levels in practice. For instance, it is much easier for the Santana do Cariri stream to sell a higher percentage of its production as monofloral honey then its counterparts due to the existence of a well-defined blossoming season for a clear honey-source flower [38].

Once the most important strategies have been identified, it is still necessary to check for the alignment of those strategies with each other towards the prioritized performance goal [38]. Alignment means the value chain stream strategies should converge to a performance goal. This implies that the streams may find it difficult to pursue strategies that aim at both production growth and local value-added at the same time. If they do pursue strategies that do not converge, suboptimal performance regarding a certain goal may be reached. For instance, the complementary feed (a production growth strategy) available at a large scale for bees may not be compatible with organic certification (a local value-added strategy). Even for the strategies that should contribute to the same performance goal, alignment should be sought. For instance, increasing the percentage of honey sold to local processor (a local value-added strategy) requires that processor to be certified as fair trade (also a local value-added strategy).

As a corollary to the results of this study, common upgrading strategies, such as those relying on product differentiation, are not the most recommended to all performance goals. Promising strategies are also frequently influenced by business scenarios. The influence of the dynamics of the business environment on promising strategies is revealed not only by changes in their importances across scenarios but also by comparison with past successful strategies. [39] showed, for instance, that adoption of technical assistance was among the top contributing strategies to production growth between 2007 and 2011, for the same honey streams. This strategy was not among the top two in any of the 2015–2020 period scenarios; it was displaced by strategies not previously pursued.

There are also strategies that rate among the top three in terms of their perceived contribution to performance in all three scenarios. These strategies are the so-called robust strategies, for which usefulness does not depend on the scenario. This is the case for ‘complimentary colony feeding’ for production growth, and ‘certification of honey as organic’ and ‘certification of honey as fair trade’ for local value-added. The common characteristic of these strategies is that they fit the traditional patterns of process (productivity improvements) and product (product differentiation) upgrading [11]. This reinforces the relevance of the process and product upgrading types because these strategies are robust across different scenarios.

The use of upgrading typologies for value chain strategies, however, should be further discussed. The two types of upgrading, product and process, for which examples of robust strategies were found in this study both relate to established upgrading forms studied in the economics of innovation [58]. These types can still be easily observed when the upgrading perspective is aggregated for networks of firms, as in a value chain stream. The other two upgrading types that were added by GVC theory in the initial debate of economic upgrading [59]–functional and inter-chain–are intrinsically related to moves by individual firms. Therefore, they deserve a closer look when the unit of analysis is a value chain stream, and not an individual firm. Functional upgrading of a stream in a certain territory reflects decisions of stream’s firms to participate more actively in additional steps of the chain. In this study, ‘honey sold to local processor’ expresses additional involvement of stream’s firms in the processing step of the chain and may be understood as a functional upgrading strategy. Inter-chain upgrading, a type of diversification of a firm, is not captured in the assessment of a given stream because this move impacts a different value chain. In the extreme, diversification of a stream (all the segments of a value chain in a territory entering another value chain) would not be a very likely alternative. In addition, among the most important strategies for both performance goals, a few could be related to the widened concept of linkage upgrading (‘resource sharing at production step’ and ‘number of hives per beekeeper’), whereas others are not yet classified as upgrading (‘technical assistance type’ and ‘technical and managerial assistance coverage’).

It is clear that the definition of upgrading would have to be expanded to include strategies that could possibly contribute to specific value chain stream goals in any business scenario. This finding supports the argument that upgrading should be viewed as searching for a “better deal” [23], to be properly defined in each situation. Value chain strategy design in development interventions is a frequent task with a variety of perspectives and frequently overlooked alternatives [60]. Rather than expanding the upgrading concept every time a newly identified strategy does not fit the most current definition of upgrading, the application of the value chain SCP framework, as shown in this study, offers an opportunity for development practitioners and value chain participants to search for promising strategies towards a performance goal–the “better deal”–in an integrated way.

In fact, extending the SCP framework to value chains brought three innovations to the value chain strategy literature: the integrated assessment of value chains provided by the relations between structure, conduct and performance; the explicit embeddedness in the territory where the segment of the value chain (the stream) is located; and the value chain stream (a network of firms composing a segment of a value chain in a territory) itself as the unit of analysis. The value chain SCP framework takes into account the concepts of governance and value distribution from GVC theory in its conduct categories. Moreover, the framework incorporates, from GPN theory, the concept of territorial embeddedness in its unit of analysis and in its structural categories, and the notion of competing geographies in its unit of analysis. Therefore, the value chain SCP framework combines the strengths [61] of leading network approaches, GVC and GPN, towards local and regional development. By doing so, and bringing a theoretical entry point, SCP, to address the competition among value chains, the value chain SCP framework contributes to re-shaping the conceptual and heuristic discussion on upgrading strategies of value chains [23, 62, 63].

The application of the value chain SCP framework in distinct configurations of structure, conduct and performance also reveals that some traditional strategy upgrading types may be more adequate just towards certain performance goals. If the goal, for instance, is to increase local value-added, product upgrading strategies may be more appropriate. *A priori*, the implementation sequence of the strategies, nonetheless, depends on the degree of the contribution of the strategies to performance, rather than on the types of the strategies. This contrasts with the GVC theory, which expects a regular trajectory of strategy adoption, starting from process upgrading, to product, then to functional, and finally, to inter-chain upgrading [58]. However, one should keep in mind that this standard trajectory described by GVC theory is expected from individual firms [64], and not from value chain streams.

In that regard, the value chain SCP framework assumes the adoption of a certain set of strategies by a stream, to be expressed as a combination of the strategies adopted by all firms in that stream. In practice, it is possible that firms in the same step of the stream follow very different, conflicting sets of strategies. Depending on the characteristics of the stream (size of territory, number of firms in each step of the stream), those differences in the firms’ strategy sets may not be sustainable, because the stream capabilities and resources would be stretched too thin. In any case, the value chain SCP framework allows for those differences within the streams, as stream strategies can be expressed in percentages of adoption of a certain strategy (for example, percentage of production certified as organic). In practice, it is also possible that firms in different steps of the same stream engage in strategies that are beneficial to one, and at the expenses of the others. Adopting conflicting, divergent strategies [65] may not be sustainable in the long run, and may cause the stream to collapse. For example, to compensate for their high-cost, outdated processing technologies, leading honey processors in a stream may decide to pay low prices to honey producers, and consequently drive them out of business. The SCP framework addresses this concern by considering vertical relations not only among firms inside and outside the stream [66], but also relations among stream members.

This study focused on the strategies that can be pursued by the value chains to improve their competitiveness and promote local development, moving beyond upgrading typologies. The value chain SCP framework is expected to be valuable for future research in the following areas: application of the value chain SCP framework to identify strategies for other value chain streams, identification of strategies for value chain streams to achieve socio-environmental goals in addition to economic goals, and the identification of structural limitations that deserve to be targeted by value chain development interventions.

## Supporting information

S1 Appendix Structural aspects of the honey business. (DOCX)

S2 Appendix Selection of stream strategies. (DOCX)

S1 Table Calculation of mean utilities and importances from ACA output for production growth under pessimistic scenario. (XLSX)

S2 Table Calculation of intraclass correlation coefficient of utilities from ACA output for production growth under pessimistic scenario. (DOCX)

S3 Table Calculation of mean utilities and importances from ACA output for production growth under realistic scenario. (XLSX)

S4 Table Calculation of intraclass correlation coefficient of utilities from ACA output for production growth under realistic scenario. (DOCX)

S5 Table Calculation of mean utilities and importances from ACA output for production growth under optimistic scenario. (XLSX)

S6 Table Calculation of intraclass correlation coefficient of utilities from ACA output for production growth under optimistic scenario. (DOCX)

S7 Table Calculation of mean utilities and importances from ACA output for local value-added under pessimistic scenario. (XLSX)

S8 Table Calculation of intraclass correlation coefficient of utilities from ACA output for local value-added under pessimistic scenario. (DOCX)

S9 Table Calculation of mean utilities and importances from ACA output for local value-added under realistic scenario. (XLSX)

S10 Table Calculation of intraclass correlation coefficient of utilities from ACA output for local value-added under realistic scenario. (DOCX)

S11 Table Calculation of mean utilities and importances from ACA output for local value-added under optimistic scenario. (XLSX)

S12 Table Calculation of intraclass correlation coefficient of utilities from ACA output for local value-added under optimistic scenario. (DOCX)
