# Supplementary material for: Beyond upgrading typologies – In search of a better deal for honey value chains in Brazil
Source: PLoS One. 2017 Jul 25;12(7):e0181391. doi: 10.1371/journal.pone.0181391 (PMC5526544; doi:10.1371/journal.pone.0181391)
Supplement: S1 Appendix — (DOCX) [file pone.0181391.s001.docx]

**S1 Appendix. Structural aspects of the honey business**

**Table A. Structural indicators and their values for the period 2007-2011, for the selected value chain streams.**

|  |  | **Value Chain Stream** | | |
| --- | --- | --- | --- | --- |
| **Category** | **Indicator** | **Limoeiro do Norte (LN)** | **Santana do Cariri (SC)** | **Picos (P)** |
| Demand behavior | World honey consumption growth 2007-2011  (% year)^a^ | 2.7% (with growing organic and fair trade segments) | | |
|  | National honey apparent consumption growth 2007-2011 (% year)^a^ | -3.2% | | |
| Concentration of clients | World market share of top 4 honey import countries  (% of volume, 2007 and 2010)^a^ | 64%, 56% | | |
|  | National market share of top 4 food retailers  (% of sales, 2011)^a^ | 50% | | |
| Rivalry intensity | World market share of top 4 honey export countries  (% of volume, 2007 and 2010)^a^ | 49%, 44% | | |
| Entry barriers | Capital and knowledge intensity^b^ | Relatively low in production | | |
| Substitute products | Existence of relevant substitute products^b^ | Sugar, glucose syrup, and other sweeteners | | |
| Institutional environment | Taxes^b^ | Tax incentives to attract processing units offered at state and national levels | | |
|  | Subsidies^b^ | Almost no direct subsidies to producers | Almost no direct subsidies to producers | Strong direct subsidies to groups of small producers |
|  | Business chamber/board/federation^b^ | Existence of honey chamber and beekeeping federation at state and national levels | | |
|  | Labor costs^b^ | Increasing cost of labor at national level | | |
|  | Quality requirements^b^ | Stricter quality requirements at both national and international levels | | |
|  | Import tariffs^a^ | Lower import tariffs to competitors from North America (by US) and Africa (by US and EU) | | |
|  | Exchange rates^a^ | Appreciation of Brazilian Real while main competitors Argentina, Turkey, Mexico, and Vietnam depreciated their currencies against US dollar | | |
| Local natural environment | Average temperature (^o^C)^a^ | 25-29 | | |
|  | Normal rainfall (mm/year)^a^ | 721-973 | | |
|  | Main bee forage sources^b^ | *Borreria verticillata, Merremia aegytia, Croton sonderianus Müll. Arg., Hyptis suaveolens* | *Serjania sp, Croton sonderianus Müll. Arg., Borreria verticillata, Hyptis suaveolens* | *Croton sonderianus Müll. Arg., Piptadenia moniliformis, Merremia aegytia, Hyptis suaveolens* |
| Local infrastructure | Road distance to export harbor Pecém/CE (km)^a^ | 253 | 538 | 560 |
|  | Access to utilities^b^ | Partial coverage of cell phone, electricity and water in some areas of the apiaries and honey houses | | |

Source: (a) [1-11]; (b) field interviews.

[1] FAO. Food and Agricultural Organization. Production and Trade, Honey, natural – 2007-2011. 2013. [Dataset]. Available from: <http://faostat.fao.org/site/342/default.aspx>. Cited 07 March 2013.

[2] IBGE. Instituto Brasileiro de Geografia e Estatística [Brazilian Institute of Geography and Statistics]. Pesquisa Pecuária Municipal, Tabela 74 – Produção de origem animal por tipo de produto, Mel de abelha, 2001 a 2011 [Municipal Livestock Research, Table 74 – Animal originated production by type of product, Bee honey, 2001-2011]. 2012. [Dataset]. Available from: [http://www.sidra.ibge.gov.br/bda/tabela/protabl.asp?c=74andz=tando=11andi=P](http://www.sidra.ibge.gov.br/bda/tabela/protabl.asp?c=74&z=t&o=11&i=P). Cited 25 November 2012.

[3] MDIC. Ministério do Desenvolvimento, Indústria e Comércio [Brazilian Ministry of Development, Industry and Foreign Trade]. AliceWeb System of Analysis of Foreign Trade Information. 2013. [Dataset]. Available from: <http://aliceweb2.mdic.gov.br/>. Cited 15 January 2013.

[4] USITC. United States International Trade Commission. Harmonized Tariff Schedule of the United States 2011. USITC Publication 4201. Washington, DC: US Government Printing Office; 2010. Available from: <http://www.usitc.gov/publications/docs/tata/hts/bychapter/1100htsa.pdf>. Cited 27 May 2013.

[5] CBI. Ministry of Foreign Affairs of the Netherlands. CBI Product Factsheet: Honey in Germany. 2011. Available from: http://www.cbi.eu/system/files/marketintel_platforms/2011_honey_in_germany.pdf. Cited 13 Jun 2013.

[6] IBGE. Instituto Brasileiro de Geografia e Estatística [Brazilian Institute of Geography and Statistics]. Cidades*@* [Cities@]. 2013. [Dataset]. Available from: http://www.ibge.gov.br/cidadesat/topwindow.htm?1. Cited 30 January 2013.

[7] INMET. Instituto Nacional de Meteorologia [Brazilian National Institute of Meteorology]. Normais Climatológicas do Brasil 1961-1990 [Brazilian Climate Normals 1961-1990]. 1992. [Dataset]. Available from: http://[www.lce.esalq.usp.br/angelocci/NORMAIS.xls](http://www.lce.esalq.usp.br/angelocci/NORMAIS.xls). Cited 25 January 2013.

[8] IPECE. Instituto de Pesquisa e Estratégia Econômica do Ceará [Ceará State Institute for Research and Economic Strategy]. Perfil Básico Municipal 2012 [Basic Municipal Profile 2012]. 2012. Available from: <http://www.ipece.ce.gov.br/publicacoes/perfil_basico/perfil-basico-municipal-2012>. Cited 25 January 2013.

[9] ABRAS. Associação Brasileira de Supermercados [Brazilian Association of Supermarkets]. Ranking Abras. 2012. Available from: <http://www.abrasnet.com.br/economia-e-pesquisa/ranking-abras/as-500-maiores/>. Cited 02 May 2013.

[10] European Commission. Online customs tariff database (TARIC). 2013. [Dataset]. Available from: http://ec.europa.eu/taxation_customs/dds2/taric/taric_consultation.jsp?Lang=enandTaric=0409000000andArea=BRandLevel=1andSimDate=20130527andGoodsText=andOrderNum=andStartPub=andEndPub=andRegulation=#. Cited 27 May 2013.

[11] FXTOP. Historical Comparison. 2012. [Dataset]. Available from: <http://fxtop.com/en/historical-exchange-rates-comparison.php>. Cited 20 November 2012.
