# Supplementary material for: Beyond upgrading typologies – In search of a better deal for honey value chains in Brazil
Source: PLoS One. 2017 Jul 25;12(7):e0181391. doi: 10.1371/journal.pone.0181391 (PMC5526544; doi:10.1371/journal.pone.0181391)
Supplement: S2 Appendix — (DOCX) [file pone.0181391.s002.docx]

**S2 Appendix. Selection of stream strategies**

**Table A. Preliminary list of strategies by value chain SCP category with number of votes per scenario and performance indicator.**

| **Category** | **Strategy** | **Votes per scenario and performance indicator*** | | | | | |
| --- | --- | --- | --- | --- | --- | --- | --- |
|  |  | **Pessimistic** | | **Realistic** | | **Optimistic** | |
|  |  | **Production Growth** | **Local Value Added** | **Production Growth** | **Local Value Added** | **Production Growth** | **Local Value Added** |
| Product/  market | Increase in honey certified as organic as % of production | 24 | 39 | 30 | 42 | 30 | 45 |
|  | Increase in honey certified as fair trade as % of production | 16 | 40 | 22 | 40 | 22 | 43 |
|  | Increase of monofloral production as % of production | 9 | 31 | 12 | 34 | 21 | 33 |
|  | Increase in honey exports as % of production | 18 | 24 | 31 | 30 | 30 | 36 |
|  | Exploitation of new bee products (e.g. propolis) | 25 | 36 | 23 | 29 | 33 | 39 |
| Pricing | Processed honey price reduction as compared to competing streams | 6 | 14 | 8 | 12 | 12 | 6 |
| Promotion | Indication of stream origin for honey products | 21 | 33 | 16 | 30 | 24 | 30 |
| Distribution channels | (no suggestion) | 0 | 0 | 0 | 0 | 0 | 0 |
| Production | Increase in the number of HACCP accredited honey house units per 100 beekeepers | 30 | 33 | 39 | 36 | 42 | 33 |
|  | Increase in the practice of migratory apiculture | 30 | 18 | 16 | 11 | 21 | 14 |
|  | Increase in the practice of bee feeding during dry season (using natural honey or complimentary feeds like soy flour) | 40 | 25 | 33 | 26 | 32 | 23 |
|  | Use of alternative vehicles to collect and transport honey to honey houses and processors | 12 | 3 | 22 | 9 | 18 | 6 |
| Product development | Movement from outside the stream to local development of honey products | 15 | 20 | 8 | 23 | 17 | 23 |
| Sourcing | Identification and exploitation of new sources of bee forages | 24 | 15 | 15 | 18 | 18 | 15 |
| Vertical linkages | Increase in % of local honey production sold to local processors^a^ | 18 | 27 | 20 | 27 | 30 | 36 |
|  | Increase in % of production from vertical or quasi-vertical integration among producers and processors^a^ | 9 | 27 | 17 | 20 | 15 | 24 |
| Horizontal linkages | Movement from associative to cooperative-type of horizontal linkage among producers | 25 | 28 | 15 | 26 | 21 | 22 |
| Network linkages | Increase in participation of stream representatives in board/chamber/federation | 21 | 15 | 21 | 17 | 24 | 15 |
| Agglomeration | Increase the number of hives per beekeeper | 12 | 15 | 24 | 20 | 36 | 24 |
| Use/quality of supporting services | Offer of specialized technical assistance | 36 | 33 | 41 | 32 | 33 | 33 |
|  | Offer of technical assistance with free distribution of hives | 13 | 16 | 18 | 16 | 19 | 19 |
|  | Increase in coverage of technical and managerial assistance | 36 | 33 | 34 | 32 | 36 | 33 |
|  | Increase in coverage of credit | 33 | 27 | 31 | 23 | 33 | 18 |

*Number of votes added for all streams. Strategies for which the number of votes is underlined were selected as belonging to the top 10.

^a^Overlapping with each other.

Source: Interview with experts and research analysis.

**Table B. Additional list of strategies by value chain SCP category with number of votes per scenario and performance indicator.**

| **Category** | **Strategy** | **Votes per scenario and performance indicator*** | | | | | |
| --- | --- | --- | --- | --- | --- | --- | --- |
|  |  | **Pessimistic** | | **Realistic** | | **Optimistic** | |
|  |  | **Production Growth** | **Local Value Added** | **Production Growth** | **Local Value Added** | **Production Growth** | **Local Value Added** |
| Product/  market | Promotion of honey in the domestic market^a^ | 9 | 3 | 6 | 0 | 6 | 0 |
|  | Export to new markets other than US and EU (e.g., Japan and Middle East) | 6 | 6 | 0 | 0 | 0 | 0 |
| Production | Recovery of original bee forage from semi-arid region (*Myracrodruon urundeuva*, *Anadenanthera colubrina*, *Mimosa caesalpiniifolia,* *Anacardium occidentale L.*); Growing of bee forage species that blossom in the dry season (*Spondias mombi,* *Spondias tuberosa,* *Prosopis juliflora,* *Anacardium occidentale L.*); Consociate beekeeping with seed oil (peanuts, castor, sunflower) and fruit growing (citrus, cashew, melon etc.)^b^ | 8 | 2 | 5 | 2 | 5 | 2 |
|  | Selection of more productive bees (genetic improvement); Systematic replacement of queen bees | 6 | 6 | 6 | 6 | 6 | 6 |
|  | Increase quality of honey extraction equipment (vertical spin extractor, automated uncapping table) | 0 | 3 | 0 | 3 | 0 | 3 |
| Use/quality of supporting services | Increase in laboratory capacity to conduct quality control analysis | 0 | 0 | 3 | 0 | 3 | 0 |
| Network linkages | Increase in the exchange of information among stream participants (e.g., fair, magazine etc.) | 3 | 6 | 3 | 6 | 3 | 6 |
| Agglomeration | Attract more material and equipment suppliers (e.g., hive manufacturers, wax honeycomb manufacturers) to the streams | 0 | 3 | 0 | 3 | 0 | 3 |

*Number of votes added for all streams. Strategies for which the number of votes is underlined were selected because they received more than three votes per scenario.

^a^Partly overlapped with ‘increase in honey exports as % of production’ in Table A, thus new resulting strategy became ‘increase in honey exports as % of processed volume’.

^b^Overlapped with ‘identification and exploitation of new sources of bee forages’ in Table A.

Source: Interview with experts and research analysis.
