# Supplementary material for: Beyond upgrading typologies – In search of a better deal for honey value chains in Brazil
Source: PLoS One. 2017 Jul 25;12(7):e0181391. doi: 10.1371/journal.pone.0181391 (PMC5526544; doi:10.1371/journal.pone.0181391)
Supplement: S2 Table — (DOCX) [file pone.0181391.s004.docx]

**S2 Table. Calculation of intraclass correlation coefficient of utilities from ACA output for production growth under pessimistic scenario**

**Descriptives**

| **Descriptive Statistics** | | | | | |
| --- | --- | --- | --- | --- | --- |
|  | N | Minimum | Maximum | Mean | Std. Deviation |
| Resp1 | 38 | -1,20 | ,94 | -,0111 | ,48311 |
| Resp2 | 38 | -,61 | ,65 | -,0357 | ,33279 |
| Resp3 | 38 | -,92 | ,50 | ,0074 | ,34221 |
| Resp4 | 38 | -,31 | ,52 | ,0405 | ,21574 |
| Resp5 | 38 | -,86 | ,58 | ,0642 | ,34962 |
| Resp6 | 38 | -,73 | ,43 | -,0230 | ,28412 |
| Resp7 | 38 | -,61 | ,66 | ,0253 | ,37136 |
| Resp8 | 38 | -,34 | ,35 | ,0022 | ,19651 |
| Resp9 | 38 | -,60 | ,60 | ,0775 | ,33287 |
| Resp10 | 38 | -,69 | ,66 | ,0322 | ,36493 |
| Resp11 | 38 | -,80 | ,50 | ,0414 | ,28433 |
| Resp12 | 38 | -,54 | ,65 | ,0077 | ,25652 |
| Resp13 | 38 | -,75 | ,77 | ,0328 | ,36287 |
| Resp14 | 38 | -,41 | ,28 | -,0319 | ,17736 |
| Resp15 | 38 | -,58 | ,63 | ,0395 | ,35624 |
| Valid N (listwise) | 38 |  |  |  |  |

**Scale: ALL VARIABLES**

| **Case Processing Summary** | | | |
| --- | --- | --- | --- |
|  | | N | % |
| Cases | Valid | 38 | 100,0 |
|  | Excluded^a^ | 0 | ,0 |
|  | Total | 38 | 100,0 |

| a. Listwise deletion based on all variables in the procedure. |
| --- |

| **Reliability Statistics** | |
| --- | --- |
| Cronbach's Alpha | N of Items |
| ,953 | 15 |

| **Intraclass Correlation Coefficient** | | | | | | |
| --- | --- | --- | --- | --- | --- | --- |
|  | Intraclass Correlation^b^ | 95% Confidence Interval | | F Test with True Value 0 | | |
|  |  | Lower Bound | Upper Bound | Value | df1 | df2 |
| Single Measures | ,577^a^ | ,464 | ,702 | 21,457 | 37 | 518 |
| Average Measures | ,953 | ,928 | ,972 | 21,457 | 37 | 518 |

| **Intraclass Correlation Coefficient** | |
| --- | --- |
|  | F Test with True Value 0^b^ |
|  | Sig |
| Single Measures | ,000 |
| Average Measures | ,000 |

| Two-way random effects model where both people effects and measures effects are random. |
| --- |
| a. The estimator is the same, whether the interaction effect is present or not. |
| b. Type C intraclass correlation coefficients using a consistency definition-the between-measure variance is excluded from the denominator variance. |
