# Supplementary material for: Beyond upgrading typologies – In search of a better deal for honey value chains in Brazil
Source: PLoS One. 2017 Jul 25;12(7):e0181391. doi: 10.1371/journal.pone.0181391 (PMC5526544; doi:10.1371/journal.pone.0181391)
Supplement: S4 Table — (DOCX) [file pone.0181391.s006.docx]

**S4 Table. Calculation of intraclass correlation coefficient of utilities from ACA output for production growth under realistic scenario**

**Descriptives**

| **Descriptive Statistics** | | | | | |
| --- | --- | --- | --- | --- | --- |
|  | N | Minimum | Maximum | Mean | Std. Deviation |
| resp1 | 38 | -1.20 | .94 | -.0111 | .48319 |
| resp2 | 38 | -.61 | .65 | -.0357 | .33279 |
| resp3 | 38 | -.92 | .50 | .0074 | .34221 |
| resp4 | 38 | -.31 | .52 | .0405 | .21574 |
| resp5 | 38 | -.86 | .58 | .0642 | .34962 |
| resp6 | 38 | -.73 | .43 | -.0230 | .28412 |
| resp7 | 38 | -.61 | .66 | .0253 | .37136 |
| resp8 | 38 | -.34 | .35 | .0022 | .19651 |
| resp9 | 38 | -.60 | .60 | .0775 | .33287 |
| resp10 | 38 | -.69 | .66 | .0322 | .36493 |
| resp11 | 38 | -.80 | .50 | .0414 | .28433 |
| resp12 | 38 | -.54 | .65 | .0077 | .25652 |
| resp13 | 38 | -.75 | .77 | .0328 | .36287 |
| resp14 | 38 | -.41 | .28 | -.0319 | .17736 |
| resp15 | 38 | -.58 | .63 | .0395 | .35624 |
| Valid N (listwise) | 38 |  |  |  |  |

**Scale: ALL VARIABLES**

| **Case Processing Summary** | | | |
| --- | --- | --- | --- |
|  | | N | % |
| Cases | Valid | 38 | 100.0 |
|  | Excluded^a^ | 0 | .0 |
|  | Total | 38 | 100.0 |

| a. Listwise deletion based on all variables in the procedure. |
| --- |

| **Reliability Statistics** | |
| --- | --- |
| Cronbach's Alpha | N of Items |
| .953 | 15 |

| **Intraclass Correlation Coefficient** | | | | | | | |
| --- | --- | --- | --- | --- | --- | --- | --- |
|  | Intraclass Correlation^b^ | 95% Confidence Interval | | F Test with True Value 0 | | | |
|  |  | Lower Bound | Upper Bound | Value | df1 | df2 | Sig |
| Single Measures | .577^a^ | .464 | .702 | 21.456 | 37 | 518 | .000 |
| Average Measures | .953 | .928 | .972 | 21.456 | 37 | 518 | .000 |

| Two-way random effects model where both people effects and measures effects are random. |
| --- |
| a. The estimator is the same, whether the interaction effect is present or not. |
| b. Type C intraclass correlation coefficients using a consistency definition. The between-measure variance is excluded from the denominator variance. |
