# Supplementary material for: Beyond upgrading typologies – In search of a better deal for honey value chains in Brazil
Source: PLoS One. 2017 Jul 25;12(7):e0181391. doi: 10.1371/journal.pone.0181391 (PMC5526544; doi:10.1371/journal.pone.0181391)
Supplement: S6 Table — (DOCX) [file pone.0181391.s008.docx]

**S6 Table. Calculation of intraclass correlation coefficient of utilities from ACA output for production growth under optimistic scenario**

**Descriptives**

| **Descriptive Statistics** | | | | | |
| --- | --- | --- | --- | --- | --- |
|  | N | Minimum | Maximum | Mean | Std. Deviation |
| Resp1 | 35 | -,96 | ,93 | -,0160 | ,42824 |
| Resp2 | 35 | -,65 | ,72 | ,0335 | ,30035 |
| Resp3 | 35 | -,75 | ,62 | -,0348 | ,33806 |
| Resp4 | 35 | -1,16 | ,90 | ,0053 | ,56822 |
| Resp5 | 35 | -,60 | ,58 | ,0306 | ,34074 |
| Resp6 | 35 | -,59 | ,67 | ,0012 | ,33115 |
| Resp7 | 35 | -,31 | ,46 | ,0678 | ,20581 |
| Resp8 | 35 | -,35 | ,55 | ,0608 | ,20496 |
| Resp9 | 35 | -,40 | ,52 | ,0606 | ,23127 |
| Resp10 | 35 | -,71 | ,75 | ,0224 | ,39685 |
| Resp11 | 35 | -,96 | ,76 | ,0181 | ,36219 |
| Resp12 | 35 | -,47 | ,46 | ,0628 | ,26779 |
| Resp13 | 35 | -1,12 | ,84 | ,0018 | ,46136 |
| Resp14 | 35 | -1,18 | ,78 | -,0185 | ,52103 |
| Resp15 | 35 | -1,16 | 1,51 | ,0771 | ,63534 |
| Valid N (listwise) | 35 |  |  |  |  |

**Scale: ALL VARIABLES**

| **Case Processing Summary** | | | |
| --- | --- | --- | --- |
|  | | N | % |
| Cases | Valid | 35 | 100,0 |
|  | Excluded^a^ | 0 | ,0 |
|  | Total | 35 | 100,0 |

| a. Listwise deletion based on all variables in the procedure. |
| --- |

| **Reliability Statistics** | |
| --- | --- |
| Cronbach's Alpha | N of Items |
| ,939 | 15 |

| **Intraclass Correlation Coefficient** | | | | | | |
| --- | --- | --- | --- | --- | --- | --- |
|  | Intraclass Correlation^b^ | 95% Confidence Interval | | F Test with True Value 0 | | |
|  |  | Lower Bound | Upper Bound | Value | df1 | df2 |
| Single Measures | ,509^a^ | ,390 | ,649 | 16,523 | 34 | 476 |
| Average Measures | ,939 | ,906 | ,965 | 16,523 | 34 | 476 |

| **Intraclass Correlation Coefficient** | |
| --- | --- |
|  | F Test with True Value 0^b^ |
|  | Sig |
| Single Measures | ,000 |
| Average Measures | ,000 |

| Two-way random effects model where both people effects and measures effects are random. |
| --- |
| a. The estimator is the same, whether the interaction effect is present or not. |
| b. Type C intraclass correlation coefficients using a consistency definition-the between-measure variance is excluded from the denominator variance. |
