# Supplementary material for: Beyond upgrading typologies – In search of a better deal for honey value chains in Brazil
Source: PLoS One. 2017 Jul 25;12(7):e0181391. doi: 10.1371/journal.pone.0181391 (PMC5526544; doi:10.1371/journal.pone.0181391)
Supplement: S8 Table — (DOCX) [file pone.0181391.s010.docx]

**S8 Table. Calculation of intraclass correlation coefficient of utilities from ACA output for value-added under pessimistic scenario**

**Descriptives**

| **Descriptive Statistics** | | | | | |
| --- | --- | --- | --- | --- | --- |
|  | N | Minimum | Maximum | Mean | Std. Deviation |
| Resp1 | 43 | -1,08 | ,88 | -,0320 | ,52155 |
| Resp2 | 43 | -,68 | ,55 | ,0483 | ,31644 |
| Resp3 | 43 | -,81 | ,88 | -,0283 | ,43707 |
| Resp4 | 43 | -,73 | ,52 | ,0263 | ,30497 |
| Resp5 | 43 | -,59 | ,56 | ,0450 | ,34029 |
| Resp6 | 43 | -,34 | ,36 | -,0008 | ,19869 |
| Resp7 | 43 | -,24 | ,25 | ,0186 | ,14753 |
| Resp8 | 43 | -,13 | ,19 | ,0588 | ,08362 |
| Resp9 | 43 | -,29 | ,47 | ,0924 | ,19553 |
| Resp10 | 43 | -,78 | ,53 | ,0093 | ,31161 |
| Resp11 | 43 | -,81 | ,57 | ,0094 | ,32028 |
| Resp12 | 43 | -,43 | ,35 | -,0146 | ,19421 |
| Resp13 | 43 | -1,01 | ,71 | ,0193 | ,40550 |
| Resp14 | 43 | -,62 | ,63 | -,0293 | ,27955 |
| Resp15 | 43 | -,59 | ,75 | ,0313 | ,37529 |
| Valid N (listwise) | 43 |  |  |  |  |

**Scale: ALL VARIABLES**

| **Case Processing Summary** | | | |
| --- | --- | --- | --- |
|  | | N | % |
| Cases | Valid | 43 | 100,0 |
|  | Excluded^a^ | 0 | ,0 |
|  | Total | 43 | 100,0 |

| a. Listwise deletion based on all variables in the procedure. |
| --- |

| **Reliability Statistics** | |
| --- | --- |
| Cronbach's Alpha | N of Items |
| ,943 | 15 |

| **Intraclass Correlation Coefficient** | | | | | | |
| --- | --- | --- | --- | --- | --- | --- |
|  | Intraclass Correlation^b^ | 95% Confidence Interval | | F Test with True Value 0 | | |
|  |  | Lower Bound | Upper Bound | Value | df1 | df2 |
| Single Measures | ,523^a^ | ,415 | ,647 | 17,439 | 42 | 588 |
| Average Measures | ,943 | ,914 | ,965 | 17,439 | 42 | 588 |

| **Intraclass Correlation Coefficient** | |
| --- | --- |
|  | F Test with True Value 0^b^ |
|  | Sig |
| Single Measures | ,000 |
| Average Measures | ,000 |

| Two-way random effects model where both people effects and measures effects are random. |
| --- |
| a. The estimator is the same, whether the interaction effect is present or not. |
| b. Type C intraclass correlation coefficients using a consistency definition-the between-measure variance is excluded from the denominator variance. |
