# Supplementary material for: Beyond upgrading typologies – In search of a better deal for honey value chains in Brazil
Source: PLoS One. 2017 Jul 25;12(7):e0181391. doi: 10.1371/journal.pone.0181391 (PMC5526544; doi:10.1371/journal.pone.0181391)
Supplement: S10 Table — (DOCX) [file pone.0181391.s012.docx]

**S10 Table. Calculation of intraclass correlation coefficient of utilities from ACA output for value-added under realistic scenario**

**Descriptives**

| **Descriptive Statistics** | | | | | |
| --- | --- | --- | --- | --- | --- |
|  | N | Minimum | Maximum | Mean | Std. Deviation |
| Resp1 | 38 | -,83 | ,90 | -,0275 | ,42080 |
| Resp2 | 38 | -,93 | ,97 | ,0201 | ,35801 |
| Resp3 | 38 | -,90 | ,78 | ,0159 | ,37035 |
| Resp4 | 38 | -,53 | ,58 | ,0220 | ,28145 |
| Resp5 | 38 | -,91 | ,62 | ,0062 | ,42389 |
| Resp6 | 38 | -,89 | ,82 | ,0272 | ,49548 |
| Resp7 | 38 | -,07 | ,15 | ,0445 | ,05539 |
| Resp8 | 38 | -,32 | ,36 | ,0204 | ,13752 |
| Resp9 | 38 | -,26 | ,40 | ,0692 | ,15408 |
| Resp10 | 38 | -,61 | ,68 | ,0486 | ,35200 |
| Resp11 | 38 | -,96 | ,70 | ,0550 | ,37922 |
| Resp12 | 38 | -,33 | ,50 | ,0346 | ,20960 |
| Resp13 | 38 | -,43 | ,45 | ,0515 | ,25998 |
| Resp14 | 38 | -,11 | ,11 | ,0026 | ,05496 |
| Resp15 | 38 | -,63 | ,75 | ,0058 | ,38344 |
| Valid N (listwise) | 38 |  |  |  |  |

**Scale: ALL VARIABLES**

| **Case Processing Summary** | | | |
| --- | --- | --- | --- |
|  | | N | % |
| Cases | Valid | 38 | 100,0 |
|  | Excluded^a^ | 0 | ,0 |
|  | Total | 38 | 100,0 |

| a. Listwise deletion based on all variables in the procedure. |
| --- |

| **Reliability Statistics** | |
| --- | --- |
| Cronbach's Alpha | N of Items |
| ,950 | 15 |

| **Intraclass Correlation Coefficient** | | | | | | |
| --- | --- | --- | --- | --- | --- | --- |
|  | Intraclass Correlation^b^ | 95% Confidence Interval | | F Test with True Value 0 | | |
|  |  | Lower Bound | Upper Bound | Value | df1 | df2 |
| Single Measures | ,556^a^ | ,443 | ,685 | 19,816 | 37 | 518 |
| Average Measures | ,950 | ,923 | ,970 | 19,816 | 37 | 518 |

| **Intraclass Correlation Coefficient** | |
| --- | --- |
|  | F Test with True Value 0^b^ |
|  | Sig |
| Single Measures | ,000 |
| Average Measures | ,000 |

| Two-way random effects model where both people effects and measures effects are random. |
| --- |
| a. The estimator is the same, whether the interaction effect is present or not. |
| b. Type C intraclass correlation coefficients using a consistency definition-the between-measure variance is excluded from the denominator variance. |
