# Supplementary material for: Beyond upgrading typologies – In search of a better deal for honey value chains in Brazil
Source: PLoS One. 2017 Jul 25;12(7):e0181391. doi: 10.1371/journal.pone.0181391 (PMC5526544; doi:10.1371/journal.pone.0181391)
Supplement: S12 Table — (DOCX) [file pone.0181391.s014.docx]

**S12 Table. Calculation of intraclass correlation coefficient of utilities from ACA output for value-added under optimistic scenario**

**Descriptives**

| **Descriptive Statistics** | | | | | |
| --- | --- | --- | --- | --- | --- |
|  | N | Minimum | Maximum | Mean | Std. Deviation |
| Resp1 | 38 | -1,00 | 1,19 | -,0243 | ,44663 |
| Resp2 | 38 | -,63 | ,69 | ,0314 | ,28994 |
| Resp3 | 38 | -1,10 | ,94 | ,0246 | ,38931 |
| Resp4 | 38 | -,62 | ,59 | ,0118 | ,29207 |
| Resp5 | 38 | -,33 | ,41 | ,0411 | ,19985 |
| Resp6 | 38 | -,64 | ,58 | -,0040 | ,34452 |
| Resp7 | 38 | -,34 | ,38 | ,0572 | ,17381 |
| Resp8 | 38 | -,10 | ,23 | ,0546 | ,10053 |
| Resp9 | 38 | -,68 | ,72 | ,0827 | ,34665 |
| Resp10 | 38 | -,62 | 1,02 | ,0405 | ,36542 |
| Resp11 | 38 | -1,07 | 1,02 | ,0160 | ,39896 |
| Resp12 | 38 | -,40 | ,43 | ,0206 | ,21497 |
| Resp13 | 38 | -,54 | ,56 | ,0533 | ,34396 |
| Resp14 | 38 | -,73 | ,78 | -,0300 | ,39526 |
| Resp15 | 38 | -,61 | ,74 | ,0676 | ,31217 |
| Valid N (listwise) | 38 |  |  |  |  |

**Scale: ALL VARIABLES**

| **Case Processing Summary** | | | |
| --- | --- | --- | --- |
|  | | N | % |
| Cases | Valid | 38 | 100,0 |
|  | Excluded^a^ | 0 | ,0 |
|  | Total | 38 | 100,0 |

| a. Listwise deletion based on all variables in the procedure. |
| --- |

| **Reliability Statistics** | |
| --- | --- |
| Cronbach's Alpha | N of Items |
| ,953 | 15 |

| **Intraclass Correlation Coefficient** | | | | | | |
| --- | --- | --- | --- | --- | --- | --- |
|  | Intraclass Correlation^b^ | 95% Confidence Interval | | F Test with True Value 0 | | |
|  |  | Lower Bound | Upper Bound | Value | df1 | df2 |
| Single Measures | ,576^a^ | ,463 | ,702 | 21,404 | 37 | 518 |
| Average Measures | ,953 | ,928 | ,972 | 21,404 | 37 | 518 |

| **Intraclass Correlation Coefficient** | |
| --- | --- |
|  | F Test with True Value 0^b^ |
|  | Sig |
| Single Measures | ,000 |
| Average Measures | ,000 |

| Two-way random effects model where both people effects and measures effects are random. |
| --- |
| a. The estimator is the same, whether the interaction effect is present or not. |
| b. Type C intraclass correlation coefficients using a consistency definition-the between-measure variance is excluded from the denominator variance. |
